# Supplementary material for: Barriers and facilitators in physical activity among youth with cerebral palsy in Sweden: A qualitative study
Source: PLoS One. 2026 Mar 31;21(3):e0346578. doi: 10.1371/journal.pone.0346578 (PMC13037959; doi:10.1371/journal.pone.0346578)
Supplement: Attachment — Submitted filename: Response to reviewers 2025-12-15.docx [file pone.0346578.s002.docx]

# Response to reviewers

Thank you for reviewing this manuscript, now named **Barriers and facilitators in physical activity among youth with cerebral palsy: A qualitative study**. You provided several useful comments that we have addressed in detail below. We believe they have improved the manuscript greatly. Page- and line numbers refer to when the track-changes are invisible.

|  | **EDITORS** |  |
| --- | --- | --- |
| 1 | Please ensure that your manuscript meets PLOS ONE's style requirements, including those for file naming. | Thank you for this reminder  -We have edited the references so that brackets are used in the text.  -Headings are adjusted according to guidelines  -Title page author information has been clarified according to PlosONE guidelines |
| 2 | You indicated that you had ethical approval for your study. In your Methods section, please ensure you have also stated whether you obtained consent from parents or guardians of the minors included in the study or whether the research ethics committee or IRB specifically waived the need for their consent. | Thank you, the following clarification is added in the beginning of the methods section: *Consent from parents or guardians is not required from the age of 15, according to the Swedish Ethical Review Authority, provided that the participants are able to understand their engagement in research and provide informed consent. All participants were deemed capable to provide informed consent. Participants were invited to share information about the study with their guardians, though this remained at their discretion, lines 161-170*. |
| 3 | We note that the grant information you provided in the ‘Funding Information’ and ‘Financial Disclosure’ sections do not match.  When you resubmit, please ensure that you provide the correct grant numbers for the awards you received for your study in the ‘Funding Information’ section | Thank you for pointing this out. The information is now matching, and grant numbers are provided where possible, however not all grants have delivered a grant number. |
| 4 | Thank you for stating the following financial disclosure: “FD, BIK: Umeå Center for Gender Studies at Umeå University; MB: the Linnea and Josef Carlsson Foundation; FD: The Queen Silvia’s Jubilee foundation 2019; and MB, BE: the Strategic Research Area Health Care Science (SFO-V), Umeå University.” Please state what role the funders took in the study.  If the funders had no role, please state: "The funders had no role in study design, data collection and analysis, decision to publish, or preparation of the manuscript."  If this statement is not correct you must amend it as needed.  Please include this amended Role of Funder statement in your cover letter; we will change the online submission form on your behalf | “*The funders had no role in study design, data collection and analysis, decision to publish, or preparation of the manuscript*” is now added to the Funding-section in the cover letter.  Funding information (and other declarations) are removed from the title page and placed only in cover letter. |
| 5 | If the reviewer comments include a recommendation to cite specific previously published works, please review and evaluate these publications to determine whether they are relevant and should be cited. There is no requirement to cite these works unless the editor has indicated otherwise. | N/A |
|  | **REVIEWER 1** |  |
| 6 | Title: A title should be concise and comprehensive to readership. It should always indicate the study design used. This is not the case with this title "**Navigating Norms and Barriers: Youth with Cerebral Palsy in Physical Education and Leisure Activities in Sweden**." Please, have a closer look at the title to make it more precise and to prompt reading, especially to people not in the field. | . The following title is now added **Barriers and facilitators in physical activity among youth with cerebral palsy: A qualitative study** |
| 7 | Introduction: What is the purpose and significance of this study? For the purpose of this study, could you explain the difference between physical education and physical leisure activities? Please indicate gaps from the literature review that this study intends to bridge. | The purpose is stated on lines 106-110 *to* ***explore the experiences of physical education and physical leisure activities among youth with cerebral palsy. Specifically, it investigates how these youths navigate contextual barriers and facilitators to participation, with particular attention to norms related to function and gender*.** It is important to explore this and give voice to youth themselves in order to shed light on inequity. This is further specified: *This knowledge is essential for understanding the underlying factors contributing to the low levels of physical activity among individuals with cerebral palsy compared to WHO recommendations. In a broader context, it may contribute to insight into determinants of societal participation, experiences of discrimination, and health lines 116-18*  Lines 54-58 clarifies discrimination as a barrier, that requires youth experiences to further explore.  Line 84-85, clarification on the need for individual experiences to exemplify inclusive physical activity.  Lines 92-95 clarifies the unequal participation in a school setting, that is supposed to be adapted for pupils to participate according to their prerequisites. Qualitative data may reveal how, which varies between individuals, context and time.  103-106 indicates the social norms potentially limiting participation, which are explored through our interviews, leading to the aim on lines 106-110.  111-119 clarifies our intent to provide a norm-critical theoretical contribution, which is less prevalent.  Physical education is clarified early in the introduction, line 45, with the addition “physical education *in school*”. It is further explained on mid page. 4, line 86-94. Physical leisure is to a different degree voluntary or depending on local and personal/family prerequisites, besides norms explored in this manuscript. Compared to physical education, physical leisure activity is vaguer. This is now clarified in data collection lines 211-212. *These terms were not specifically defined but rather the participants were allowed to describe their views and experiences.* |
| 8 | - What are the relevant controversies in this field from your literature review? | The introduction describes social and societal injustice based on physical function and other factors, partly seen in our paper preceding this study—e.g. that the Swedish law of education is not followed since adapted physical education does not seem sufficient for participation. Hence, a need for the youths’ voices and experiences for understanding these patterns. While some studies have previously been made with children/youths regarding particpation, our contribution is empirical as well as theoretical and addresses foremost the norms and structures impacting on participation, targeting physical leisure activity and physical education specifically. Please see clarifications in the previous review question, above. |
| 9 | Methodology: Could you justify the choice of methodology for this study? | Yes, we added a short sentence to the data analysis section 234-36: *This methodology provides a systematic as well as flexible framework to analyse data, allowing latent interpretation meanwhile staying close to data (the participants’ narratives)*. |
| 10 | - This sentence "Their physical function ranged from very limited ability to voluntary movement to very small motor limitations." is difficult to understand. For clarity purposes, I suggest you reformulate this sentence in a way that a lay person can grasp what you want to communicate. | Thank you for pointing this out. We do want our text to be accessible to lay persons. The text now reads: “Participants’ physical abilities varied widely—from very limited movement, requiring help to steer an electric wheelchair, to only minor difficulties, such as a slight limp or slower running speed.” (lines 173-175) |
| 11 | - Lines 173-176 is not clear. Please clarify. | We agree that this is unclear. The section now reads “*Inclusion criteria were being 15–18 years of age, having a diagnosis of cerebral palsy and being capable of providing informed consent to research participation. Moreover, while not an explicit criterion, the use of language or communication techniques not familiar to the interviewer or requiring training, was discussed with the recruiting care providers as a potential risk of misinterpretation of the participants*.” Lines 184-188 |
| 12 | - Participants between 15 and 18 years in this study are minors and parental consent is an obligation. Was parental consent needed for any of the participants? The authors have not explicitly indicated anywhere in this section whether or not parental/guardian consent was duly obtained. | Parental consent for persons aged 15 or older is not an obligation according to the Swedish Ethical Review Authority, if participants are capable of providing informed consent to research. This is clarified in the beginning of the methods section 161-170. |
| 13 | - Were the interviews done with the participants alone or in the presence of a parent/guardian? What measures were put in place to maintain comfort of the participants, considering their special conditions, during interviews? Was there any occasion that necessitated discontinuing the interview because a participant did not feel well or just stressed to continue? If yes, how was the situation handled? | This is an important question. One participant was accompanied by a school assistant at the interview, as they preferred that. This is clarified in data collection 204-206. The others preferred to participate one on one, in a place agreed upon, which is explained. Two participants showed signs of becoming tired towards the end of the interviews but still wanted to proceed when this was addressed. This is also clarified 208-210. |
| 14 | - Patients with cerebral palsy are individuals with different skills and capabilities. What were the criteria for selecting participants for this study? | This is now clarified (lines 184-188): Inclusion criteria were: being 15–18 years of age, having a diagnosis of cerebral palsy and being capable of providing informed consent to participate in research. Besides this, we wished for heterogeneity in our sample to obtain a variety of experiences, which we achieved in terms of e.g. gender and function but not e.g. ethnic background, which we address in methodological discussion. |
| 15 | - What steps were taken to ensure reliability and triangulation of data? | Reliability may be included in the term trustworthiness, stated in data analysis on p. 11 lines 244-54, but cannot be ensured as it would in statistics, due to the subjective and reflexive nature of qualitative methods. The way we used triangulation for trustworthiness is described in this paragraph, i.e. that the co-authors read parts of the data at different points of the analysis and discussed and compared the coding of the content to the first author’s. Minor editing was made to the text for clarity. |
| 16 | - It is not enough to state "Ethical approval was obtained". What institutional body granted the approval? | Information on ethics has now been added to the methods section Lines 161-70. This was previously not disclosed due to our wrongful presumption of a blinded review process. |
| 17 | Results and discussion: - Some quotes from the participants and interviewer lack clarity and are too fragmented. For example, lines 392-396 are not comprehensible, especially to someone not familiar with the findings of the study. These quotes can't be reproduced because of the way they have been presented to the reader. Please go through the manuscript and ensure that the quotes make meaning, standing alone | Thank you for this comment. We have made minor revisions throughout the results section for comprehension. Quotes are not deleted but clarified—either within them or in the text attaching to them—in order for them to be comprehensible in their context. However, it cannot be expected that individual quotes can be reproduced when performing individual interviews with semi-structured questions. Nevertheless, quotes should provide clarity and verify the authors’ interpretation of the data, which in turn could be reproduced in overall meaning. We hope you find our revisions of the results, particularly around the quotes, helpful for clarity. |
| 18 | - Where are the quotes to show how or what participants said in lines 404-406? Also from lines 407-446 there is not a single quote from any participant. If this section is combined results and discussion, I expect that the discussion should be aligned to the findings from the study, stating the participants perceptions and feelings. | We appreciate your thorough comment. We chose not to add a quote to lines 404-406 (final paragraph of category 2) as there was no data suitable to form a comprehensible quote that would provide clarity to the analysis. Please note that this does not equal non-empirical analysis.  The lines 407-446 refer to the discussion section of category 2, hence, no quotes. We have added the word “discussion” to the three discussion sections for clarity. |
| 19 | - Why is "Methodological considerations" part of the results and discussion section? Please explain. | You are correct in your comment. The heading level is now adjusted for clarity, into level 1—same level as results/discussion |
|  | **REVIEWER 2** |  |
| 20 | The authors have taken great care to consider the ethics of talking with youth with cerebral palsy. Their decision to not share their data publicly is sound to protect the confidentiality of participating youth, and they have offered the option of contacting the authors for access. This study is not one focused on replicability, in my view, it was more to understand the lived experiences of youth with cerebral palsy in participating in school sports. To that end, the need to look at the data would only be to understand the coding process/codes. The examples provided in the article do a good job of showing how the codes were created. | Thank you, we appreciate your clear interpretation of our view on data sharing and confidentiality and we fully agree with it.  We will clarify the table according to your comment below. |
|  | It might be helpful to provide examples from each of the 3 categories in Table 1 (line 221 on page 10) since the data is not publicly available. Other than that minor suggestion, it was a well designed study with thoughful analysis. | Thank you for your positive feedback and advice. Additional examples are added to table 1 concerning the remaining two categories. |
|  | Additional edits: | Brief clarifications are made in methodological discussion and conclusion to clarify our points and emphasize our focus on norms/discrimination. |
