# Supplementary material for: Barriers and facilitators in physical activity among youth with cerebral palsy in Sweden: A qualitative study
Source: PLoS One. 2026 Mar 31;21(3):e0346578. doi: 10.1371/journal.pone.0346578 (PMC13037959; doi:10.1371/journal.pone.0346578)
Supplement: Attachment — Submitted filename: Response to reviewers_2_20260206.docx [file pone.0346578.s003.docx]

# Response to reviewers

Thank you for reviewing this manuscript, now named **Barriers and facilitators in physical activity among youth with cerebral palsy: A qualitative study in Sweden**. Your useful comments are addressed in detail below. We believe they have improved the manuscript greatly. Page- and line numbers refer to when the track-changes are invisible.

|  | **REVIEWER 1** |  |
| --- | --- | --- |
| 6 | The title (comment 6) is more descriptive and understandable but should indicate where the study was done, in this case Sweden. | Thank you, this is now corrected. The following title is now used **Barriers and facilitators in physical activity among youth with cerebral palsy: A qualitative study in Sweden** |
| 18 | Your response to comment 18 "We chose not to add a quote to lines 404-406 (final paragraph of category 2) as there was no data suitable to form a comprehensible quote that would provide clarity to the analysis. Please note that this does not equal non-empirical analysis" does not address the comment. It is not what you choose to add or not. Your study data should provide evidence to draw conclusions from. If "there was no suitable data to form a comprehensive quote", then why make any statement that can't be substantiated from the interviews that you conducted? Please revisit your interviews to make an informed decision on what to add or not. | Thank you for your thorough comment. The text section is revised (p. 20, lines 441-5), and a brief quote is added to exemplify the analysis. I hope this contributes with clarity. **The responsibility the teenagers took upon themselves also indicated a sense that they had a limited right to complain about their situation. As one boy expressed it, comparing himself to others: *“I know that some people are worse off, sort of”* (Participant 4, 17 years). Consequently, some described feeling that they ought to be content when they received support, regardless of its quality.** |
|  | **REVIEWER 2** |  |
| 1 | You have addressed all of the reviewer feedback effectively, strengthening the paper's methodology and discussion. | Thank you for your time and your previous comments and advice |
