# Supplementary material for: Barriers and facilitators in physical activity among youth with cerebral palsy in Sweden: A qualitative study
Source: PLoS One. 2026 Mar 31;21(3):e0346578. doi: 10.1371/journal.pone.0346578 (PMC13037959; doi:10.1371/journal.pone.0346578)
Supplement: Attachment — Submitted filename: Response to reviewers_3_20260312.docx [file pone.0346578.s004.docx]

# Response to reviewers

Thank you for re-reviewing this manuscript, now named **Barriers and facilitators in physical activity among youth with cerebral palsy in Sweden: A qualitative study**. The title is now improved.

|  | **REVIEWER 1** |  |
| --- | --- | --- |
|  | Thank you for taking time to address my comments. However, the title still needs to be rephrased. should read "Barriers... cerebral palsy in Sweden: a qualitative study". The study is about participants living in Sweden and not qualitative study in Sweden. Refer to your previous study: Degerstedt F, Björklund M, Keisu B-I, Enberg B. Unequal physical activity among children with cerebral palsy in Sweden—A national registry study. | Thank you for your thorough revision. For clarity I below include the previous correspondence referring to the title. It is now corrected according to your latest suggestion in bullet point 4 below, and it also aligns with journal guidelines. Thank you for helping refine the precision of the title, I hope you find it improved in clarity.   1. Original title: **Navigating Norms and Barriers: Youth with Cerebral Palsy in Physical Education and Leisure Activities in Sweden**   Comment: Title: A title should be concise and comprehensive to readership. It should always indicate the study design used. This is not the case with this title "Navigating Norms and Barriers: Youth with Cerebral Palsy in Physical Education and Leisure Activities in Sweden." Please, have a closer look at the title to make it more precise and to prompt reading, especially to people not in the field.   2. Revision 1: **Barriers and facilitators in physical activity among youth with cerebral palsy: A qualitative study**   Comment**:** The title (comment 6) is more descriptive and understandable but should indicate where the study was done, in this case Sweden**.**   3. Revision 2: **Barriers and facilitators in physical activity among youth with cerebral palsy: A qualitative study in Sweden**   Comment: Thank you for taking time to address my comments. However, the title still needs to be rephrased. should read "Barriers... cerebral palsy in Sweden: a qualitative study". The study is about participants living in Sweden and not qualitative study in Sweden. Refer to your previous study: Degerstedt F, Björklund M, Keisu B-I, Enberg B. Unequal physical activity among children with cerebral palsy in Sweden—A national registry study.   4. Revision 3: **Barriers and facilitators in physical activity among youth with cerebral palsy in Sweden: a qualitative study** |
